# The Influence of Precipitation Regimes and Elevated CO_2_ on Photosynthesis and Biomass Accumulation and Partitioning in Seedlings of the Rhizomatous Perennial Grass *Leymus chinensis*

**DOI:** 10.1371/journal.pone.0103633

**Published:** 2014-08-05

**Authors:** Zhuolin Li, Yuting Zhang, Dafu Yu, Na Zhang, Jixiang Lin, Jinwei Zhang, Jiahong Tang, Junfeng Wang, Chunsheng Mu

**Affiliations:** 1 Key Laboratory of Vegetation Ecology, Ministry of Education, Institute of Grassland Science, Northeast Normal University, Changchun, China; 2 Key Laboratory of Saline-alkali Vegetation Ecology Restoration in Oil Field Ministry of Education, Alkali Soil Nature Environmental Science Center, Northeast Forestry University, Harbin, China; University of Connecticut, United States of America

## Abstract

*Leymus chinensis* is a dominant, rhizomatous perennial C_3_ species in the grasslands of Songnen Plain of Northern China, and its productivity has decreased year by year. To determine how productivity of this species responds to different precipitation regimes, elevated CO_2_ and their interaction in future, we measured photosynthetic parameters, along with the accumulation and partitioning of biomass. Plants were subjected to combinations of three precipitation gradients (normal precipitation, versus normal ± 40%) and two CO_2_ levels (380±20 µmol mol^-1^,760±20 µmol mol^-1^) in controlled-environment chambers. The net photosynthetic rate, and above-ground and total biomass increased due to both elevated CO_2_ and increasing precipitation, but not significantly so when precipitation increased from the normal to high level under CO_2_ enrichment. Water use efficiency and the ratio of root: total biomass increased significantly when precipitation was low, but decreased when it was high under CO_2_ enrichment. Moreover, high precipitation at the elevated level of CO_2_ increased the ratio between stem biomass and total biomass. The effect of elevated CO_2_ on photosynthesis and biomass accumulation was higher at the low level of precipitation than with normal or high precipitation. The results suggest that at ambient CO_2_ levels, the net photosynthetic rate and biomass of *L. chinensis* increase with precipitation, but those measures are not further affected by additional precipitation when CO_2_ is elevated. Furthermore, CO_2_ may partly compensate for the negative effect of low precipitation on the growth and development of *L. chinensis.*

## Introduction

In the grassland ecosystems of the eastern Eurasian steppes, *Leymus chinensis* is a typical perennial C_3_ grass with rhizomatous propagation that is distributed widely in areas including the Russian Baikal, the northern and eastern parts of Mongolia, the North China Plain, and the Inner Mongolian plateau of China [1], [2]. It is a dominant plant species in those relatively dry areas due to its tolerance of drought and saline-alkaline soils [3]. It is also an economically and ecologically important forage grass in Northern China because it is rich in protein, minerals, carbohydrates, and is palatable to many large herbivores. Recently, the area of grassland on the steppes has been decreasing due to the effects of human disturbances, including poor land use management, overgrazing, and climate change [4]. As a consequence, grassland productivity (dominated by the productivity of *L. chinensis*) has been reduced severely.

Atmospheric carbon dioxide (CO_2_) concentrations have been increasing globally at an unprecedented rate [5]–[7]. In particular, regional climate models predict that climatic changes induced by CO_2_ will exacerbate the dryness of the semiarid region of China [8], [9].

The ecosystems of China's arid and semiarid regions are driven mainly by precipitation, which is limiting and is therefore a key determinant of vegetation productivity [10]. Net primary productivity is positively correlated with precipitation [11] – [13]. In general, mild or moderate water stress will decrease the transpiration rate (*E*) of plants by reducing stomatal conductance (*g_s_*), and it will also decrease the net photosynthetic rate (*P_n_*) [13]. Severe drought will damage the photosynthetic system and lead to lower productivity. In many regions with low precipitation, plants are smaller and have relatively smaller leaf areas, but they have more roots to absorb nutrients and water in order to maintain normal growth patterns [14]. Therefore, considerable evidence shows that plant will increase the allocation of biomass to their root systems when water or nutrients are limiting [15]. In contrast, high water content in soils or fully submerged conditions typically lead to a decline in biomass allocation to roots [16].

Elevated CO_2_ generally decreases *g_s_* and *E*, stimulates *P_n_* and increases net primary production [17] – [20]. Elevated CO_2_ has been shown to enhance production in many grassland ecosystems, such as those in Switzerland and New Zealand, and Kansas tallgrass prairie and Colorado shortgrass steppe in the United States [21]. However, plants with different photosynthetic pathways have responded differently to elevated levels of CO_2_. Increased CO_2_ enhances the response of C_3_ plants but not C_4_ plants, because the ambient CO_2_ concentration is enough to satisfy the needs of the unique photosynthetic pathway in C_4_ plants [22], [23]. An indirect effect of elevated CO_2_ is improving water use efficiency (*WUE*), which can sustain plant growth and development to some degree during dry periods [24] – [26]. As most plants respond to elevated CO_2_ with an increase in photosynthesis and biomass, at least in the short term, the allocation of biomass to roots will increase in order to enable better access to nutrients [16], [27] – [28]. However, Nowak et al. [29] suggested that the allocation to below-ground biomass may not increase under elevated CO_2_ levels, and the impact on stem and leaf biomass allocation is ambiguous. Overall, there is no clear pattern regarding the effect of elevated CO_2_ on biomass allocation in plants [16], [30].

In semiarid grassland ecosystems, water is believed to regulate plant responses to elevated levels of CO_2_ in the air; this relationship is fundamental, as CO_2_ and precipitation are essential factors that determine plant growth, development and function. Therefore, the interaction between precipitation regime and CO_2_ level is even more important for driving plant growth and development in semiarid regions. Evidence shows that plant growth and productivity are stimulated more by elevated CO_2_ during water stress than under well-watered conditions [31] – [33]. However, some research suggests that plant responses to elevated CO_2_, in terms of growth and productivity, are constrained by drought [34] – [36]. The conflicting conclusions depend mainly on the severity and duration of aridness, as well as the plant species under study.

Earlier studies have considered the effect of water stress or elevated CO_2_ on growth of *L. chinensis*. However, to our knowledge few studies have addressed that subject by simulating the gradient involved in real precipitation regimes. The objective of this study was to assess changes in the growth of a dominant grassland species in response to such environmental variation; this is important to understand because overall community properties are strongly influenced by the characteristics of dominants. Thus, we measured photosynthetic parameters, and biomass accumulation and partitioning in *L. chinensis,* along a gradient of three precipitation levels (normal precipitation and normal ±40%) and two CO_2_ levels (380±20 µmol mol^−1^ and 760±20 µmol mol^−1^), to investigate the strategy that this drought-tolerant grass uses to adapt to current environmental stresses.

Typically, elevated levels of CO_2_ and precipitation stimulate the growth of C_3_ species, and rising atmospheric CO_2_ improves the efficiency of water use by plants, possibly helping to alleviate the impacts of drought via water-saving effects [37]. We therefore predicted that: (1) there would be a synergistic effect of increased precipitation and CO_2_ levels on photosynthesis and biomass accumulation of *L. chinensis*; (2) elevated CO_2_ would compensate partly for the negative effect of low precipitation on the response variables; (3) biomass allocation would be altered under elevated CO_2_ and different precipitation regimes.

## Materials and Methods

### Ethics Statement

No specific permissions were required to conduct the field research described, because the Songnen Artificial Grassland Ecological Research Station belongs to Northeast Normal University. The field site is not privately owned or protected in any way, and the study did not involve endangered or protected species. Activities followed the research guidelines of the University.

### Soil and plant cultures

Soil and seeds of the “yellow-green” ecotype of *L. chinensis* were obtained from the Field Station, which is part of the Institute of Grassland Science, Jilin Province, in northeastern China (123°44′E, 44°44′N, 167 m elevation). This region has a semiarid, continental monsoon climate with a frost-free period of about 140 d. Annual mean temperature is 6.4°C, and annual mean precipitation is 361.6 mm (2000–2011), most of which (70%) occurs during the summer months of Jun-Aug. The ecosystem's main soil type is mollisol.

The soils were sieved through 2-mm mesh to remove roots and other visible debris, mixed well, and then put into plastic pots with an inside diameter of 19 cm, and height of 14 cm. Each pot was filled with 3.3 kg dry soil. The total nitrogen, organic carbon content, EC and pH of the mollisol soil were 6.8%, 0.3%, 180 µs cm^−1^ and 8.63, respectively. To produce enough seedlings of uniform size, on 1 Sep 2012 about 20 seeds were sown in each of 24 plastic pots for a total of 480 seeds, at 15 d after sowing the seedlings were thinned to 10 per pot.

**Figure 1:**
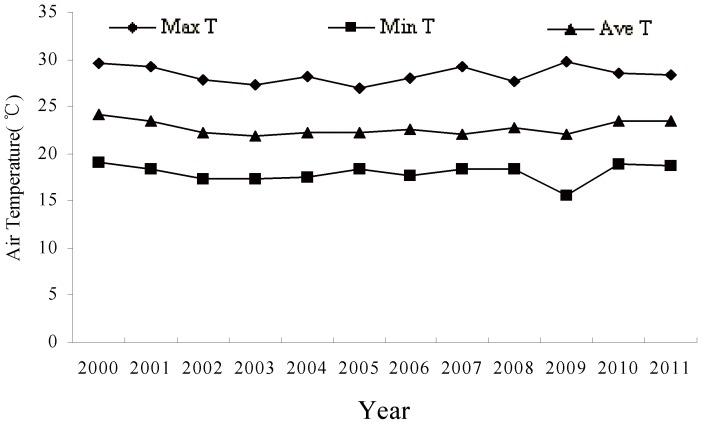
The average maximum, minimum and mean air temperatures on the semi-arid Songnen Grassland during summers (Jun-Aug) from 2000 to 2011. Data were collected by the Meteorological Bureau of Changling County, China.

**Figure 2:**
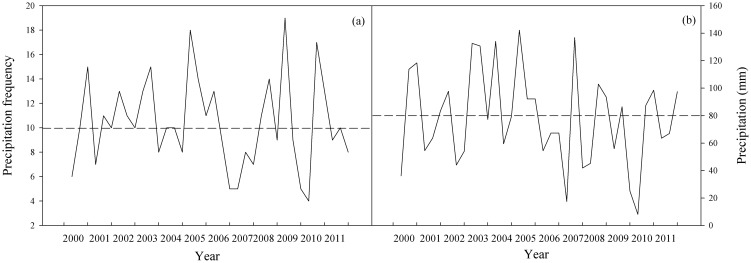
The frequency and amount of rainfall precipitation in summer (Jun-Aug) from 2000 to 2011. The average monthly precipitation was ∼80 mm. Data were collected by the Meteorological Bureau of Changling County, China.

Experimental pots were placed in the phytotron (LT/ACR-2002 Phytotron System, E-Sheng Tech., Beijing, China) at Northeast Normal University in Changchun. In the phytotron, high-stress sodium lamps (Philips) with photosynthetically active radiation provided light at a rate of 350 µmol^−2^ S^−1^ for 14 h per day. The relative humidity was maintained at 40–60%, and the temperature regime was 22°C from 5:30–8:30, 25°C from 8:30–11:30, 28°C from 11:30–14:30, 25°C from 14:30–17:30, 22°C 17:30–19:30 and 18°C from 19:30–5:30. Air temperature in each chamber was monitored and adjusted every 10 s throughout the day and night, and maintained within ±1°C of treatment set points. The pots were irrigated with 240 mL of water every 3 d (equivalent to 8 mm of precipitation, totaling 80 mm per month), with the soil water content maintained at 50–60% of field capacity. The length of diurnal/nocturnal periods was chosen to mimic the typical length of daylight hours in the Songnen grassland during summer. Temperatures matched the minimum, maximum, and average summer temperatures from 2000–2011 in the same region (Meteorological Bureau of Changling County, China, the grown site of *L. chinensis*) (Fig. 1 and Fig. 2*b*).

### Precipitation regime and CO_2_ treatments

Precipitation and CO_2_ treatments commenced one month after sowing, and the experiment spanned three months. The pots of seedlings were placed randomly (and equally) into two controlled-environment growth chambers. One chamber was randomly assigned to ambient CO_2_ at 380±20 µmol mol^−1^. The other chamber was assigned to an elevated level of 760±20 µmol mol^−1^, because atmospheric CO_2_ concentrations are predicted to double by the end of this century, which might have an important influence on the productivity of grasslands dominated by *L. chinensis* and their community structure. The CO_2_ was supplied from a tank and delivered through 0.64 cm tubing, and the concentrations were monitored every 5 s and adjusted every 10 s throughout the day and night.

In each CO_2_ chamber, pots were assigned to one of three precipitation levels: normal, high (normal +40%), and low (normal –40%). Total monthly precipitation was supplied in ten equal amounts to represent the normal monthly water level (i.e., 8 mm of precipitation every 3d). This was based one the region's average amount and frequency of precipitation during the summers of 2000–2011 (Fig. 2*a* and *2b*). Based on data for the Songnen grassland over the last 12 years, we found that precipitation in wet and dry years measured 40% higher or lower than the average, so we used that variation to define our experimental water regime.

In order to ensure that each plant experienced similar light conditions, the pot positions were randomly changed every 3 d during the treatment. Further, because there were no chamber replicates in this study, we rotated the treatments between the two chambers every 2 weeks, changing the environmental settings so that all pots were handled as similarly as possible during the experiment.

### Leaf gas exchange

Leaf CO_2_ exchange parameters were measured with an LI–6400 gas exchange system (LI–6400XT, Li–Cor, Inc., Lincoln, NE, USA) on the youngest available fully expanded leaves (3 leaves per pot, 4 pots per treatment) before sampling.

*P*_n_, *g*_s_, and *E* were measured with a LiCOR red/blue LED light source in a standard 2×3 cm chamber. The photosynthetically active radiation (PAR) was set at 350 µmol^−2^ S^−1^ to equal the light of the phytotron, and the reference CO_2_ concentration was maintained at 380±20 µmol mol^−1^ in the control and 760±20 µmol mol^−1^ in the elevated CO_2_ growth chamber using CO_2_ control modules. Samples were allowed to acclimate for a few minutes until the *P*_n_ stabilized and the coefficient of variation was below 0.5. WUE (defined as mmol of net CO_2_ uptake per mol of H_2_O lost) was derived from the ratios of *P*_n_ to *E*.

### Soil water content and biomass

After three months of treatment, we collected plants from 4 pots per treatment, carefully washed the soil from the roots in running water, and separated the plants into leaves, stems, roots and rhizomes. A soil sample of about 10 g was collected from each pot and placed in an aluminum can for measurement of soil water content (SWC). Plant parts and soils were dried to a constant mass at 65°C and weighed.

### Statistical analysis

Data were analyzed as a split-plot design with CO_2_ being the main plot and precipitation regime being the subplot (SPSS Inc, Chicago, IL, USA). Values for the photosynthetic parameters, biomass accumulation and biomass partitioning were tested for normality and homogeneity, and were transformed appropriately if necessary. For each parameter, the difference between the two CO_2_ treatments was determined with a t-test, and differences among precipitation treatments were determined with a one-way ANOVA. Levels of *P*<0.05 were considered to be significant.

## Results

### Soil water content

**Figure 3:**
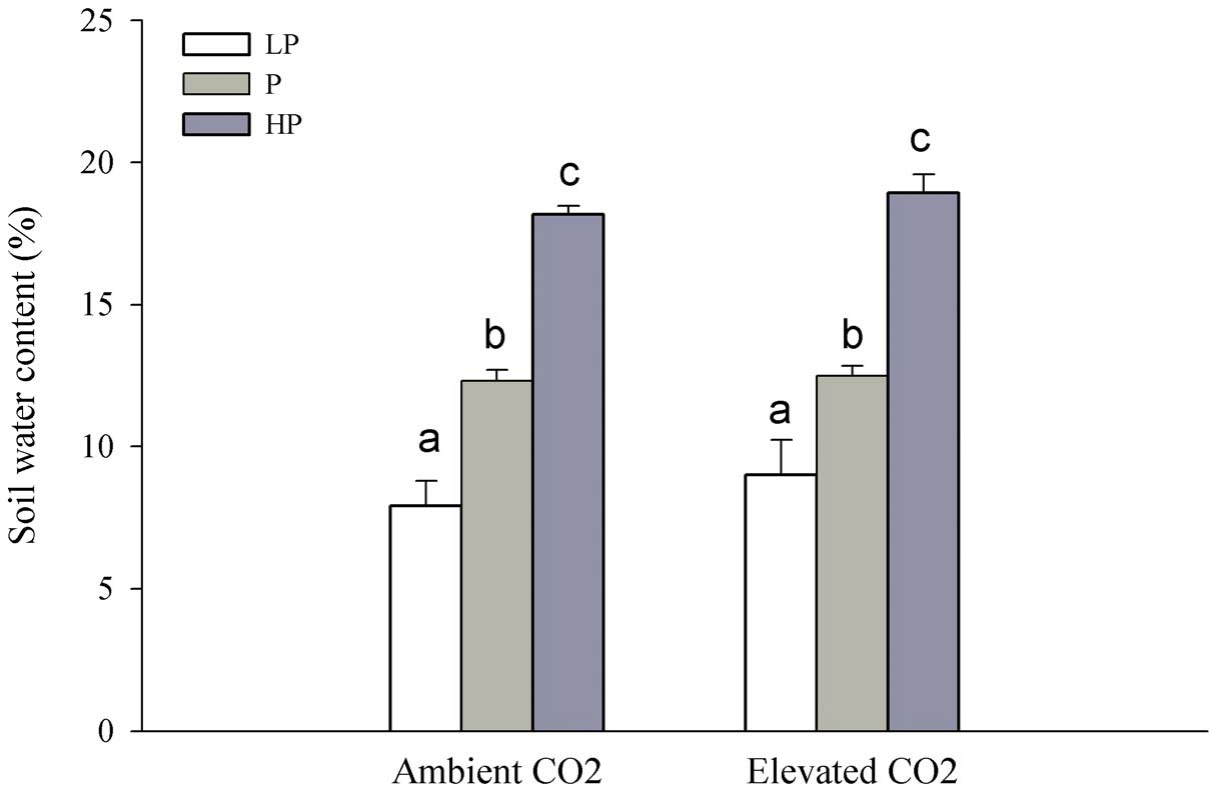
Effect of precipitation on soil water content under two CO_2_ concentrations. LP, P, HP represents low precipitation (−40%), normal precipitation and high precipitation (+40%), respectively. Different letters indicate a significance difference among levels of precipitation (*P*<0.05).

Soil water content increased significantly with precipitation at both CO_2_ levels (*P*<0.001), and showed a weak increase in elevated CO_2_ although it was not statistically significant (*P* = 0.074). Soil water content was not significantly affected by the interaction of precipitation and elevated CO_2_ (Table 1, Fig. 3).

**Table 1:** Analysis of variance to assess the impacts of precipitation, CO_2_, and their interaction on soil water content, photosynthetic parameters, biomass accumulation and biomass allocation in the grass species *L. Chinensis*.

	Source of variation					
	CO_2_		Precipitation		CO_2_×Precipitation	
	F	P	F	P	F	P
Soil water content	3.844	0.074	304.408	**<0.001**	0.642	0.543
	**Photosynthetic parameters**					
*P*n	400.232	**<0.001**	12.992	**<0.001**	7.771	**<0.001**
*C*i	741.983	**<0.001**	18.223	**<0.001**	4.985	**<0.001**
*g*_s_	290.268	**<0.001**	222.368	**<0.001**	48.112	**<0.001**
*E*	1116.369	**<0.001**	363.371	**<0.001**	144.112	**<0.001**
*WUE*	400.232	**<0.001**	12.992	**<0.001**	7.771	**<0.001**
	**Biomass**					
Above-ground	21.731	**<0.01**	34.501	**<0.001**	0.534	0.599
Below-ground	0.068	0.798	115.944	**<0.001**	20.072	**<0.001**
Total biomass	10.132	**<0.01**	72.149	**<0.001**	2.901	0.094
Root/shoot	27.192	**<0.001**	1.308	0.306	11.576	**<0.01**
	**Biomass allocation**					
Leaf	1.254	0.285	3.550	0.062	2.089	0.167
Stem	13.809	**0.003**	9.317	**0.004**	1.532	0.255
Root	26.459	**<0.001**	19.799	**<0.001**	1.467	0.269
Rhizome	1.046	0.327	5.690	**0.018**	0.596	0.566

Note:Data are significant at *P*<0.05 level (bolded values).

### Gas exchange parameters

**Figure 4:**
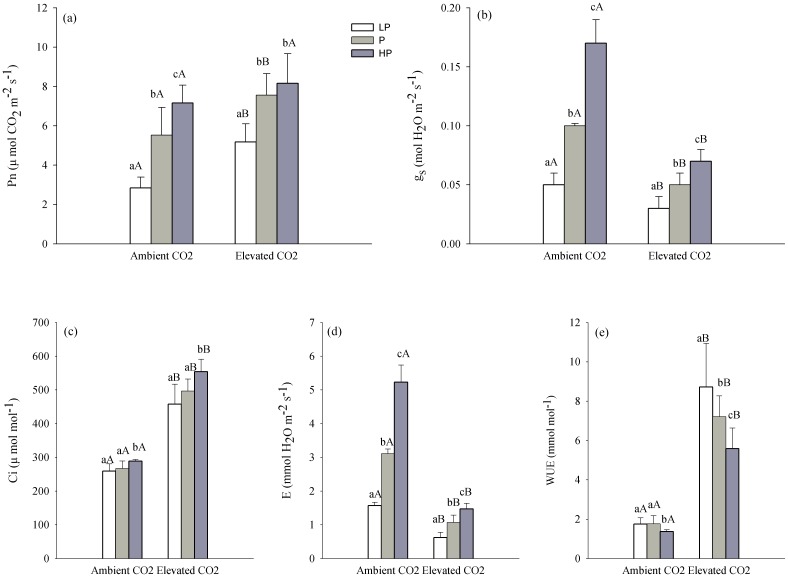
Effect of precipitation on photosynthetic rate (*P*_n_), stomatal conductance (*g*_s_), intercellular CO_2_ concentration (*C*i), transpiration rate (*E*) and water use efficiency (*WUE*) of *Leymus chinensis* under two CO_2_ concentrations. Different lower-case letters indicate a significant difference among different levels of precipitation and different capital letters indicate a significant difference between the two CO_2_ levels (*P*<0.05). LP, P, HP represents low precipitation (−40%), normal precipitation and high precipitation (+40%), respectively.

Both precipitation regime and elevated CO_2_ had a significant influence on the *P*_n_, *g*_s_, *C*_i_, *E,* and *WUE* (Table 1). Ambient CO_2_, *P*_n_, *g*_s_ and *E* increased significantly with increasing precipitation (Fig. 4*a, b, d*), whereas *WUE* showed a significant decline under high precipitation, compared to under low and normal precipitation (*P*<0.001) (Fig. 4*e*). Photosynthetic characters of *L. chinensis* under elevated CO_2_ responded in a similar way as under ambient CO_2_. However, *P*_n_ under normal and high precipitation exhibited no significant response, although it was higher in plants grown under high precipitation compared to normal levels (Fig. 4*a*). *WUE* decreased as precipitation increased at the elevated level of CO_2_ (Fig. 4*e*).

Elevated CO_2_ significantly increased *P*_n_ — the greatest change was under low precipitation, at 82.4%, with a change of 36.7% and 14.0% at the normal and high levels of precipitation. There was no difference under high precipitation (*P*>0.05) (Fig. 4*a*). *C*_i_ responded in a similar way as *P*_n_ (Fig. 4*c*). Compared to in the ambient CO_2_ conditions, *g*_s_ decreased significantly by 40%, 50% and 58.8% at the low, normal and high levels of precipitation, respectively (*P*<0.001) (Fig. 4*b*), *E* decreased by 60.5%, 65.6%, and 71.9% (*P*<0.001) (Fig. 4*d*), whereas *WUE* increased by 398.3%, 307.3% and 308% (*P*<0.001) (Fig. 4*e*). These results indicated that elevated CO_2_ had a greater effect on the *P*_n_ and *WUE* of seedlings, especially under drier conditions, likely to compensate for the negative effects induced by drought.

### Biomass accumulation and allocation

Overall, precipitation had a significant influence on above-ground, below-ground, and total biomass. Elevated CO_2_ also had a significant influence on above-ground biomass, total biomass and root: shoot ratio. Their interaction, however, affected only below-ground biomass and root: shoot ratio (Table 1). At ambient CO_2_, the above-ground, below-ground, and total biomass of individuals increased significantly with increasing precipitation (Table 2; *P*<0.05). Under elevated CO_2_, low precipitation significantly decreased individual biomass, while high precipitation caused a slight increase in above-ground biomass. Notably, that enhancement caused by increasing precipitation was not observed under elevated CO_2_ when precipitation increased from the normal to high level.

**Table 2:** Effect of precipitation on biomass of *L. Chinensis* under two CO_2_ concentrations.

CO_2_ concentration	Index (g plant^−1^)	Precipitation (mm)		
		Low(LP)	Normal(P)	High(HP)
Ambient CO_2_ (380 µmol mol^−1^)	Above-ground biomass	0.27±0.02aA	0.43±0.01bA	0.48±0.03bA
	Below-ground biomass	0.36±0.01aA	0.66±0.03bA	0.72±0.01cA
	Total biomass	0.64±0.04aA	1.09±0.03bA	1.21±0.03cA
	Root: shoot ratio	1.34±0.06aA	1.54±0.06aA	1.50±0.06aA
Elevated CO_2_ (760 µmol mol^−1^)	Above-ground biomass	0.35±0.03aA	0.57±0.04bB	0.59±0.01bA
	Below-ground biomass	0.49±0.01aB	0.65±0.03bA	0.62±0.02bB
	Total biomass	0.84±0.03aB	1.22±0.07bA	1.21±0.04bA
	Root: shoot ratio	1.42±0.06aA	1.15±0.04bB	1.06±0.06bB

Note: Different lower-case letters indicate a significant difference among precipitation levels and different capital letters indicate a significant difference between the two CO_2_ levels (*P*<0.05). Low/High precipitation were defined as normal precipitation +/−40%.

Elevated CO_2_ increased above-ground biomass by 29.6%, 32.6% and 22.9%, compared to plants with a similar watering regime (from low to high) at ambient CO_2_, although only the effect under normal precipitation was significant (*P*<0.05). Below-ground biomass increased significantly at low precipitation and decreased at high precipitation under CO_2_ enrichment compared to ambient CO_2_ conditions (*P*<0.05). Therefore, elevated CO_2_ significantly increased individual total biomass at low precipitation, but this enhancement was not seen under high precipitation conditions. Furthermore, elevated CO_2_ significantly decreased the root: shoot ratio under normal and high precipitation conditions (*P*<0.05). Thus, elevated CO_2_ advanced above-ground vegetation growth under favorable water conditions, and increased below-ground biomass under drought conditions, presumably to facilitate water absorption.

**Figure 5:**
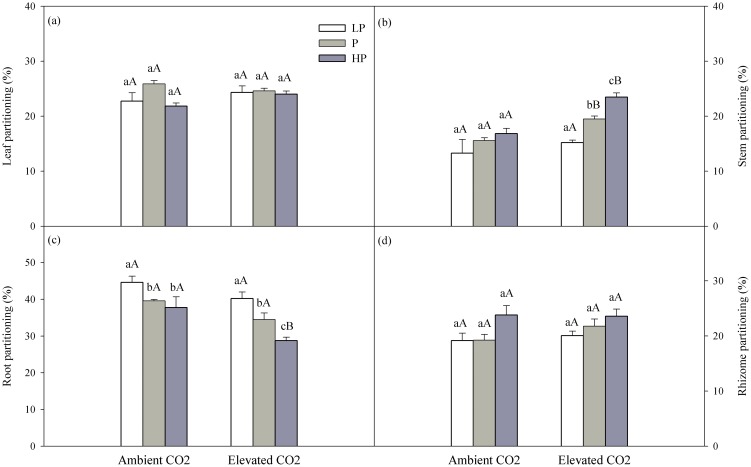
Effect of precipitation on biomass allocation among plant parts under two CO_2_ concentrations for *Leymus chinensis*. Different lower-case letters indicate a significant difference among different precipitation levels, and different capital letters indicate a significant difference between the two CO_2_ levels (*P*<0.05). LP, P, HP represents low precipitation (−40%), normal precipitation and high precipitation (+40%), respectively.

Both precipitation and CO_2_ had a significant influence on biomass allocation to stems and roots, whereas no significant effect of interaction was detected between precipitation and CO_2_ on biomass allocation (Table 1). Stem biomass allocation increased with precipitation at both CO_2_ levels, but only elevated CO_2_ caused a significant effect (Fig. 5; *P*<0.05). Biomass allocation to roots responded in opposite directions: it tended to be higher under low precipitation at both CO_2_ levels (*P*<0.05), and under normal and high precipitation it was significantly lower at elevated CO_2_ (*P*<0.05), but there was no effect with ambient CO_2_.

At a given level of precipitation, elevated CO_2_ significantly increased biomass allocation to stems under normal and high precipitation compared to ambient CO_2_ (*P*<0.05), and decreased biomass allocation to roots under high precipitation conditions (*P*<0.05).

## Discussion

In arid and semiarid regions, precipitation is the key variable that affects the growth and development of vegetation, and it can also affect plant metabolism and signal transduction. As elevated levels of atmospheric CO_2_ are the main cause of global climate change, we investigated the interaction of precipitation and CO_2_ levels to provide a more comprehensive assessment of how current environmental changes may be affecting plant growth in a semiarid region. Substantial research efforts have demonstrated that elevated CO_2_ causes an increase in *P*_n_ and the accumulation of biomass in plants, while drought has the opposite effect [13], [20], [32] – [33], [38] – [40]. However, results concerning the interaction between precipitation regime and elevated CO_2_ have been ambiguous [41] – [43]. The results of our research support our predictions 2 and 3, that increasing precipitation and elevated CO_2_ improve biomass and *P*_n_ of *L. chinensis*, and moreover, those effects were greater under low precipitation conditions than under high precipitation. Those findings indicated that elevated CO_2_ could lead to increased soil water content and compensate for the negative effect of drought on the growth of this grass species. The allocation of biomass among the parts of individual plants had changed too. Furthermore, soil EC decreased with increasing precipitation and elevated CO_2_, but pH did not change much (from 8.15 to 8.6; data not shown). Ma and Liang [44] suggested that seed germination and seedling growth of *L. chinensis* are highest when pH is between 8.0 and 8.5. Consequently, elevated CO_2_ and precipitation effects mediated through decreased salinity and alkalinity is expected to have minor effects in this study. Intriguingly, we found that *P*_n_ did not respond to elevated CO_2_ under high precipitation conditions, which, contrary to our first prediction, means that elevated CO_2_ and increasing precipitation did not have a synergistic effect on *P*_n_. Therefore, biomass accumulation in *L. chinensis* did not change much in response to elevated CO_2_ when precipitation increased by 40%.

### Additional precipitation does not enhance biomass accumulation and net photosynthetic rate under CO_2_ enrichment

*L. chinensis* is highly tolerant to drought, and the water holding capacity of soils where it grows site on the Songnen grassland is 50–60% in the summer months (Jun-Aug). In this study, the water holding capacity of soils under high precipitation conditions climbed to 80% or more, so above-ground biomass per plant increased weakly when precipitation increased from the normal to high level (Table 2). A similar trend was observed in *P*_n_.

Elevated CO_2_ can directly increase the carboxylation efficiency of C_3_ species, or induce stomatal closure and then limit the rate of transpiration indirectly, causing an increase in *WUE* [31], [40]. Elevated CO_2_ would not increase above-ground biomass and *Pn* in the grass species that we studied, because decreased transpiration at elevated levels of CO_2_ would provide little additional benefit in increased soil moisture [45]. Xu *et al.* [14] also found that mild and moderate drought had no significant influence on the biomass of *L. chinensis*, whereas mild drought (field capacity: 60–65%) stimulates the accumulation of biomass, consistent with the pattern observed in our study. Furthermore, some researchers have found that the below-ground biomass of grasses is suppressed by elevated CO_2_ [46]; that trend occurred in our system in the high precipitation treatment, leading to no change in total biomass, which may be because the soil moisture levels were adequate for growth so more roots were not needed to increase water absorption. This may be a typical growth strategy and ecological adaptation of *L. chinensis* in the semiarid Songnen grassland. Therefore, we infer that extra precipitation is essentially redundant, as it does not augment biomass accumulation and *P*_n_ under higher CO_2_ levels due to the high resistance of this species to soil water stress.

### Elevated CO_2_ might partly compensate for the negative effect of drought on net photosynthetic rate and biomass

In line with our predictions, elevated CO_2_ seemed to partly compensate for the negative effect of drought on *Pn* and biomass of *L. chinensis*. Our results support the notion that some herbaceous species are more stimulated by elevated CO_2_ under water stress than under well-watered conditions [23], [24], [31], [33], [45].

Elevated CO_2_ reduced *g*_s_, and the effect of elevated CO_2_ on the percent change of *g*_s_ was smallest at the low precipitation level (Fig. 4*b*). This maybe because both drought and elevated CO_2_ caused a decline in *g*_s_ to limit *E*, and elevated CO_2_ has a minimum effect on *g*_s_ under low precipitation in order to maintain levels of photosynthesis. However, although elevated CO_2_ increased the substrate concentration under high precipitation conditions, *g*_s_ decreased quickly to restrain absorption of CO_2_. Therefore, at the elevated CO_2_ level, the increasing percent change of *P*_n_ for grass plants grown under low precipitation conditions was considerably higher than for those grown under high precipitation conditions.

In general, in the arid and semiarid grassland, *WUE* is also an important factor that stimulates primary productivity [47], especially in years with low precipitation. We found that elevated CO_2_ significantly improved *WUE* (Table 1, Fig. 4*e*), and slightly increased soil water content under low precipitation conditions although the change was not significant (Table 1, Fig. 3*a*). In the Kansas Tallgrass Prairie experiment, volumetric soil water content was generally higher in elevated CO_2_ plots than under ambient levels of the gas, mainly during periods when precipitation limited normal plant growth due to drought [21]. This phenomenon occurred in a healthy dry ecosystem. *WUE* improved ∼3–4 fold when CO_2_ concentration was doubled, which means that the amount of water plants needed to fix one unit of CO_2_ decreased by ∼3–4 fold [48]. This is important in regions experiencing drought and dry soils due to low precipitation. Therefore, although the effect of elevated CO_2_ on plant growth is limited under low precipitation conditions, elevated CO_2_ still partly compensated for the negative effect of drought on *P*_n_ of *L. chinensis.*

The increase in *P*_n_ indicates that the ability of plants to fix carbon can be enhanced. From low to high precipitation levels, elevated CO_2_ increased total biomass per plant by 31.3%, 11.9% and 0%, respectively, as compared to at ambient CO_2_ levels. The trend for *P*_n_ was similar. Furthermore, root: shoot ratios increased at low precipitation, yet decreased at normal and high precipitation, which is consistent with the findings of other studies [23], [34], [35]. Plants still suffer water stress even if elevated CO_2_ leads to higher *WUE* and an enhanced ability of soils to maintain water, therefore, plants will increase their below-ground biomass to absorb more water and nutrients. The increase of root: shoot ratio may be the best strategy for plants to adapt to water stress [14]. However, some other studies demonstrate that plants have greater stimulation owing to elevated CO_2_ under well-watered conditions than under drought [34] – [36]. The differences among these results may be due to the varying resistance of plants to drought, as well as different drought scales and other variation in the precise conditions studied. Our results indicate that elevated CO_2_ could partly compensate for the negative effects of drought on *P*_n_ and biomass of *L. chinensis.*

### Biomass allocation

Most archetypal vascular plants have leaves that fix carbon, stems that provide mechanical support and a hydraulic pathway, and roots that absorb nutrients and water [16]. Rhizomes function as storage organs in many clonal plants. Allocating biomass differently among these organs enables plants to balance growth and adapt environmental changes [49]. In our study, low precipitation significantly increased the allocation of biomass to roots under two levels of CO_2_, effectively enabling plants to absorb more water and nutrients and have more above-ground vegetation growth, which is in line with results of many other studies [14], [16].

We showed that elevating the environmental concentration of CO_2_ also altered the allometric relationships of biomass among plant tissues. Under normal and high precipitation conditions, elevated CO_2_ significantly increased the biomass allocation to stems, but decreased allocation to roots (Table 1, Fig. 5*b, c*). Plants allocate more biomass to stems in order to facilitate competition for light and the acquisition of carbon [17]. However, much evidence shows that elevated CO_2_ generally decreases the nitrogen concentration in leaves. Thus, it is possible that a plant allocates more biomass to roots to enable increased uptake of nutrients in order to sustain increases in biomass [16]. In contrast, a plant may regulate its root morphology and physiology rather than allocating more biomass to its roots when soil water and nutrients are abundant [50], [51]. Furthermore, biomass allocation was not affected by elevated CO_2_, as has been widely observed in managed grasslands [52], [53]. The differing results are likely due to differences in the conditions being studied, such as other environmental factors, plant species, developmental stages, etc.

In summary, our results suggest that the biomass of *L. chinensis* increases with precipitation levels at ambient levels of CO_2_. However, further precipitation is redundant, in that it does not augment biomass accumulation or the net photosynthetic rate of this grass species, and in fact it decreases biomass allocation to roots under conditions of CO_2_ enrichment. In effect, our experiments show that elevated CO_2_ may partly compensate for the negative effect of low precipitation on the growth and development of *L. chinensis.*
